# Female Bias in Systemic Lupus Erythematosus is Associated with the Differential Expression of X-Linked Toll-Like Receptor 8

**DOI:** 10.3389/fimmu.2015.00457

**Published:** 2015-09-08

**Authors:** Gabrielle McDonald, Nicholas Cabal, Augustin Vannier, Benjamin Umiker, Raymund H. Yin, Arturo V. Orjalo, Hans E. Johansson, Jin-Hwan Han, Thereza Imanishi-Kari

**Affiliations:** ^1^Department of Integrative Physiology and Pathobiology, Tufts University, Boston, MA, USA; ^2^Biosearch Technologies, Inc., Petaluma, CA, USA; ^3^Merck Research Laboratories, Palo Alto, CA, USA

**Keywords:** SLE, TLR8, X-inactivation, neutrophils, IFN-I

## Abstract

Systemic lupus erythematosus (SLE) is a chronic autoimmune disease characterized by the production of anti-nuclear antibodies. SLE is one of many autoimmune disorders that have a strong gender bias, with 70–90% of SLE patients being female. Several explanations have been postulated to account for the severity of autoimmune diseases in females, including hormonal, microbiota, and gene dosage differences. X-linked toll-like receptors (TLRs) have recently been implicated in disease progression in females. Our previous studies using the 564Igi mouse model of SLE on a *Tlr7* and *Tlr9* double knockout background showed that the presence of *Tlr8* on both X chromosomes was required for the production of IgG autoantibodies, *Ifn-I* expression and granulopoiesis in females. Here, we show the results of our investigation into the role of *Tlr8* expression in SLE pathogenesis in 564Igi females. Female mice have an increase in serum pathogenic anti-RNA IgG2a and IgG2b autoantibodies. 564Igi mice have also been shown to have an increase in neutrophils *in vivo*, which are major contributors to *Ifn-*α** expression. Here, we show that neutrophils from C57BL/6 mice express *Ifn-*α** in response to 564 immune complexes and TLR8 activation. Bone marrow-derived macrophages from 564Igi females have a significant increase in *Tlr8* expression compared to male-derived cells, and RNA fluorescence *in situ* hybridization data suggest that *Tlr8* may escape X-inactivation in female-derived macrophages. These results propose a model by which females may be more susceptible to SLE pathogenesis due to inefficient inactivation of *Tlr8*.

## Introduction

Systemic lupus erythematosus (SLE) is a chronic autoimmune disease characterized by the production of anti-nuclear antibodies (1). Like many other autoimmune disorders, SLE is more prevalent in females, with 70–90% of SLE patients being female (2). The basis for the gender bias in SLE and other autoimmune diseases is poorly understood. Hormonal differences, changes in microbiota, and X-linked gene dosage are all being investigated as potential driving factors.

Autoantibodies mediate many of the pathologies associated with SLE by forming immune complexes (IC) and depositing in various tissues, promoting tissue damage. It has been shown that autoantibodies also cause developmental defects in the offspring born to female SLE patients (3). Female patients have been shown to have an increase in miscarriages during pregnancy (4, 5). Furthermore, offspring born to female SLE patients have an increased incidence of cognitive disorders compared to healthy controls (6–11). It has been reported that certain maternal anti-DNA antibodies are able to cross the placenta during pregnancy and cause neuronal excitotoxicity in female offspring (7, 12–14). These antibodies cross-react with a fetal neuronal glutamate receptor that has differential timing of expression in male and female embryos, which accounts for the preferential female fetal death (14). Fully understanding the cause of the female bias of SLE is critical to preventing the production of such pathogenic antibodies.

Several immune-linked genes are located on the X chromosome and have been implicated in SLE pathogenesis, including endosomal toll-like receptors (TLRs) 7 and 8 (15). Endosomal TLRs 7, 8, and 9 are intracellular nucleic acid sensors that play varying roles in the development of SLE. TLR9 contributes to the production anti-DNA antibodies (16), but it was also revealed to act as a negative regulator of disease pathogenesis (17–19). TLR7 is largely thought to promote anti-RNA autoantibody production (20–25). However, *Tlr7*-deficiency in mice does not completely eliminate anti-nuclear antibody production (22, 24). It was recently reported that a deficiency of both *Tlr7* and *Tlr8* is required to eliminate anti-nuclear antibody production (15), suggesting that TLR8 is a significant contributor. TLR8 can also induce granulopoiesis in the bone marrow and *Ifn-I* expression in neutrophils in female mice (15). Human SLE patients have increased circulating granulocytes and increased IFN-I production (26, 27). IFN-I signaling is known to be involved in the development of SLE-like symptoms (28, 29).

Interestingly, SLE-like pathology, including autoantibody production, increased granulopoiesis and increased *Ifn-I* expression, are alleviated in female mice deficient in one copy of X-linked *Tlr8* (15). The phenotype of these females closely resembles that of their male counterparts, suggesting that gene dosage, not hormonal influences, may contribute the female bias of SLE. To investigate the role of TLR8 in SLE pathogenesis, we use the 564Igi mouse model of SLE. 564Igi mice have knock-in genes at the immunoglobulin (Ig) heavy (H) and light (L) chain loci that encode for an anti-RNA antibody. These mice develop SLE-like symptoms, including autoantibodies, increased granulopoiesis, increased IFN-I production, and glomerulonephritis (20). There is also a female bias in this mouse model, closely resembling human disease. 564Igi mice were found to have an increase in monocyte and neutrophil populations, both of which contribute to the increased IFN-I production (30). Recognition of IFN-I by neutrophils leads to the upregulation of FcγRIV (30). FcγRIV is the activating Fc receptor for IgG2a and IgG2b IC. The ratio of FcγRIV to FcγRIIb, the inhibitory receptor for IgG2a and IgG2b IC, determines the threshold of activation for the cell by IgG antibodies. A shift in the ratio of the receptors can make the cell more or less sensitive to activating stimuli. Thus, in 564Igi mice, expanded neutrophil and monocyte populations produce IFN-I and increase the FcγRIV:FcγRIIb ratio in neutrophils, which may make cells more susceptible to activation by IC. One potential mechanism to induce granulopoiesis in 564Igi mice is through the production of autoantibodies, which could be the initiating factor in neutrophil activation. However, autoantibody production is not sufficient for either granulopoiesis or *Ifn-I* expression in 564Igi mice (15). Therefore, an alternative mechanism must exist to promote the expansion of neutrophils, *Ifn-I* expression, and autoantibody production. Autoantibody production in 564Igi mice is known to depend on TLR7 and TLR8 (15, 20), and expression of *Tlr8* on both X chromosomes is necessary to promote SLE-like symptoms in 564Igi mice (15). Furthermore, SLE-like symptoms in 564Igi mice have a female bias. Therefore, we sought to examine the role of TLR8 in SLE pathogenesis.

Here, we show that female mice have elevated levels of pathogenic IgG autoantibodies and that *Ifn* expression in neutrophils is mediated by immune complex formation and TLR8 activation. Female mice also have significantly increased *Tlr8* expression in bone marrow-derived macrophages (BMDM), which is likely due to escape of X-inactivation. These results describe a novel mechanism of SLE pathogenesis specific for females through inefficient X-inactivation of X-linked TLR8.

## Materials and Methods

### Mice

All experiments with mice were performed in accordance with the regulations of and with the approval off the Tufts/TMC IACUC (protocol B2012-50). Creation of the 564Igi mice was previously described (20) and they were bred in house. All 564Igi mice are homozygous for both the IgH and IgL knock-in genes unless otherwise indicated. C57BL/6 and *Fc*γ*RIIb^−/−^* mice were purchased from Jackson Laboratories. *Tlr8^−/−^* mice were generously gifted by Dr. D. Golenbock and Dr. R. Gazzinelli at the University of Massachusetts Medical School, Worcester, MA, USA with the permission of Dr. R. Flavell at Yale University, New Haven, CT, USA.

### Anti-RNA/DNA ELISas

Nunc MaxiSorb flat-bottom ELISA plates (ThermoFisher 442404) were coated with 0.1% w/v poly-l-lysine solution (Sigma P8920) for 4 h at room temperature for anti-RNA ELISA or 1% protamine sulfate diluted in PBS for 30 min at room temperature for anti-DNA ELISA. The solution was then removed and 50 μg/mL of yeast RNA (Ambion AM7120G) or calf-thymus DNA (Invitrogen) diluted in 1× borate buffer (100 mM boric acid, 25 mM sodium borate, 75 mM sodium chloride) was added and incubated at 4°C overnight. Plates were washed 3× in 0.1%Tween/borate and then blocked with 5% goat serum/0.05% Tween/0.1% sodium azide/borate for anti-RNA ELISA or 1% BSA/0.1% sodium azide/borate for anti-DNA ELISA for 2 h at room temperature. Plates were washed 3× in 0.1% Tween/borate and then serum samples or cell culture supernatants were added using serial dilutions in blocking buffer and incubated at 4°C overnight. Plates were washed 3× in 0.1% Tween/borate and antibodies were detected with 1 μg/mL alkaline phosphatase (AP)-conjugated goat-anti-mouse IgG2a/IgG2b antibodies (Southern Biotech) diluted in blocking buffer. Plates were washed 5× in 0.1% Tween/borate and developed with 1 mg/mL AP substrate *p*-nitrophenyl phosphate (Life Technologies) diluted in ELISA buffer (0.1 M glycine, 1 mM zinc chloride, 1 mM magnesium chloride). The optical density (O.D.) was read at 405 nm.

### Flow cytometry

Cells were stained for flow cytometry according to standard procedures (20). Neutrophils were stained with anti-CD11b-Alexa488 and anti-Ly6C-PE or anti-Ly6G-PE (Biolegend). Macrophages were stained with anti-F4/80-Alexa647 (Biolegend). Fluorescently labeled antibodies were generally used at 1 μg/mL.

### Cell culture

Purified neutrophils were sorted by flow cytometry and cultured in 10% FCS RPMI media supplemented with penicillin/streptomycin, l-glutamine, 2-mercaptoethanol, amphotericin, sodium pyruvate, and non-essential amino acids for 16 h in the presence of CLO75 (1 mg/mL), CLO97 (1 mg/mL), or 4 h in the presence of purified 564 immune complex (0.01 μg/mL). BMDM were grown by culturing whole bone marrow cell suspensions in 20% FCS RPMI media supplemented with penicillin/streptomycin and 25% L cell line culture supernatant for 7 days.

### Quantitative real-time PCR

RNA was isolated from cells by TRIzol incubation (Life Technologies 15596) followed by chloroform extraction. All quantitative real-time PCR (RT-qPCR) experiments were performed using two serial dilutions of RNA and iScript Reverse Transcription Supermix for RT-qPCR from Bio-Rad (170-8841). Triplicate cDNA samples were used for the amplification of *β-actin*, *Ifn-*α*6, Tlr7*, and *Tlr8* using commercially available FAM primer probes (Life Technologies) and a Bio-Rad IQ5 quantitative PCR system. Gene expression was normalized to *β-actin* expression based on a standard curve.

### RNA fluorescence *in situ* hybridization

Bone marrow-derived macrophages were cultured as described above and sent to Biosearch Technologies, Petaluma, CA, USA. Stellaris^®^ RNA fluorescence *in situ* hybridization (FISH) was performed as recommended by the manufacturer and described in (31).

All probe sets were manufactured by Biosearch and labeled as follows: Xist, Quasar^®^ 670; *Eif2s3x* introns, Quasar 570; *Tlr7* exons, CalFluor Red 610, *Tlr7* introns, Quasar 570; *Tlr8* exons, Quasar 570. *Tlr8* introns, CalFluor Red 610. Cells fixed and hybridized with combinations of probe sets were imaged on a widefield Nikon fluorescence microscope and images with specific RNA signals were processed in FIJI (Image J).

### Statistical analysis

The *p* values were calculated using one-way ANOVA for all analyses, followed by the Tukey multiple comparison test (Prism GraphPad Software). For all figures, *p < 0.05, **p < 0.01, ***p < 0.001.

## Results

### Female mice have increased pathogenic anti-RNA IgG autoantibodies compared to males

**Figure 1:**
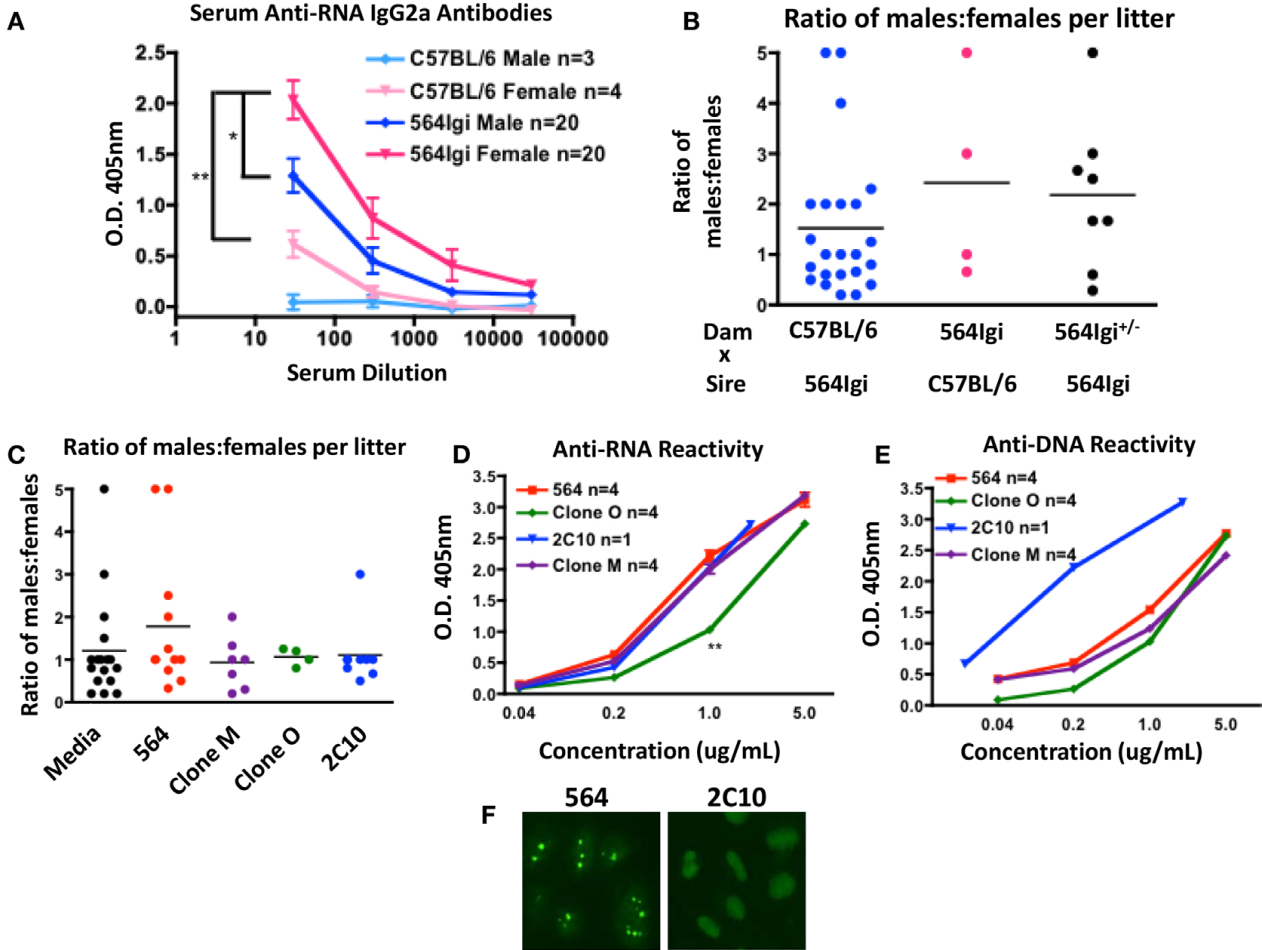
**564Igi females have more circulating pathogenic IgG autoantibodies**. **(A)** Anti-RNA antibodies were detected using and isotype-specific anti-RNA ELISA. Shown is the mean O.D. 405 nm ± SEM at various serum dilutions. The number of mice is shown in the key. **(B)** Shown is the ratio of male:female offspring born to the dam in the indicated breeding pair. Each data point represents a single litter. The average ratio is shown with a horizontal line. **(C)** Pregnant C57BL/6 dams were given introperitoneal injections with 1.5 μg of the indicated antibody or media control at approximately embryonic days 13–15. Shown is the ratio of male:female offspring in each litter. Each data point represents a single litter. The average ratio is shown with a horizontal line. **(D,E)** The mean **(D)** anti-RNA and **(E)** anti-DNA reactivities of the indicated antibodies were tested by ELISA. Shown is the mean O.D. 405 nm ± SEM at the indicated antibody concentrations. The number of replicates is shown in the key. **(F)** Purified 564 and 2C10 antibodies were tested for anti-nuclear reactivity by HEp-2 staining. Shown are the staining representative staining patterns of at least four independent experiments.

The production of autoantibodies is the characteristic symptom of SLE and mediates many of the pathologies associated with disease. To begin investigating the gender bias of SLE in 564Igi mice, we measured the RNA reactivity of serum antibodies in male and female mice. Female 564Igi mice have significantly more anti-RNA IgG2a antibodies than male 564Igi mice (Figure 1A).

It has been reported that certain maternal anti-nucleic acid antibodies can cause preferential female fetal loss by neuronal excitotoxicity (7, 12–14). To examine the effect of the 564 antibody on fetal development, we counted the number of live male and female offspring born to mothers with various genotypes. C57BL/6 females yield litters with approximately 1:1 ratio of males:females when bred to 564Igi males (Figure 1B, blue), suggesting that 564Igi males have little effect on fetal development. 564Igi females, on the other hand, yield litters with an elevated male:female ratio when bred to C57BL/6 males (Figure 1B, pink). 564Igi females are poor breeders that often take far longer to get pregnant than C57BL/6 females and often give birth to still-born offspring. In addition, 564Igi females are more likely to suffer from dystocia during pregnancy compared to C57BL/6 females. As a result, we have few data points from 564Igi females and no conclusive data from 564Igi females bred to 564Igi males. 564Igi females heterozygous for the 564 IgH and IgL knock-ins (564Igi^±^) also yield litters with elevated male:female ratios (Figure 1B, black), although there is more variability across litters.

To validate that the 564 antibody itself is mediating pathogenesis, we transfected HEK293T cells with DNA encoding the unmutated 564 antibody or mutated variants (Clone M and Clone O). Culture supernatant containing 1.5 μg of antibody or an equivalent volume of supernatant from an untransfected cell culture (Media) was then injected into C57BL/6 pregnant females. An equivalent amount of purified 2C10 anti-DNA antibody was also injected. We then counted the number of male and female offspring in each litter. The 564 antibody caused an increase in the ratio of males:females in each litter compared to the media control injection (Figure 1C, red). Clone M, Clone O, and 2C10 had no effect on the number of female offspring (Figure 1C, purple, green, and blue). The RNA and DNA reactivity of the injected antibodies was determined by ELISA (Figures 1D,E). The 2C10 antibody shows reactivity to both DNA and RNA by ELISA, however, reactivity toward DNA is much stronger. The artificial nature of the ELISAs performed here may cause some degree of cross-reactivity among anti-nucleic acid antibodies. HEp-2 staining clearly distinguishes 2C10 as a DNA-specific antibody and 564 as an RNA-specific antibody (20) (Figure 1F).

A subset of anti-DNA antibodies have been reported to cause preferential female fetal loss, as measured by similar procedures (12, 14). However, the 2C10 antibody clearly has no effect on the number of male and female offspring born in each litter, and therefore is unlikely to be considered in those classes of anti-DNA antibodies. Clone O shows reduced anti-RNA reactivity compared to the 564 antibody (Figure 1D), which may explain the normal ratio of males:females born to dams injected with Clone O. Interestingly, dams injected with Clone M also yielded litters with normal male:female ratios even though the RNA reactivity of Clone M is equivalent to that of the 564 antibody (Figure 1D). The mutations in Clone M may not affect RNA reactivity, but may affect antibody reactivity to neuronal receptors *in vivo*, resulting in normal litters despite RNA reactivity. Together, these data reflect the increased prevalence of miscarriages among female SLE patients and also implicate some anti-RNA antibodies as contributors to pathogenesis in females.

### 564 immune complexes induce neutrophils to expand and express *Ifn-α6*

**Figure 2:**
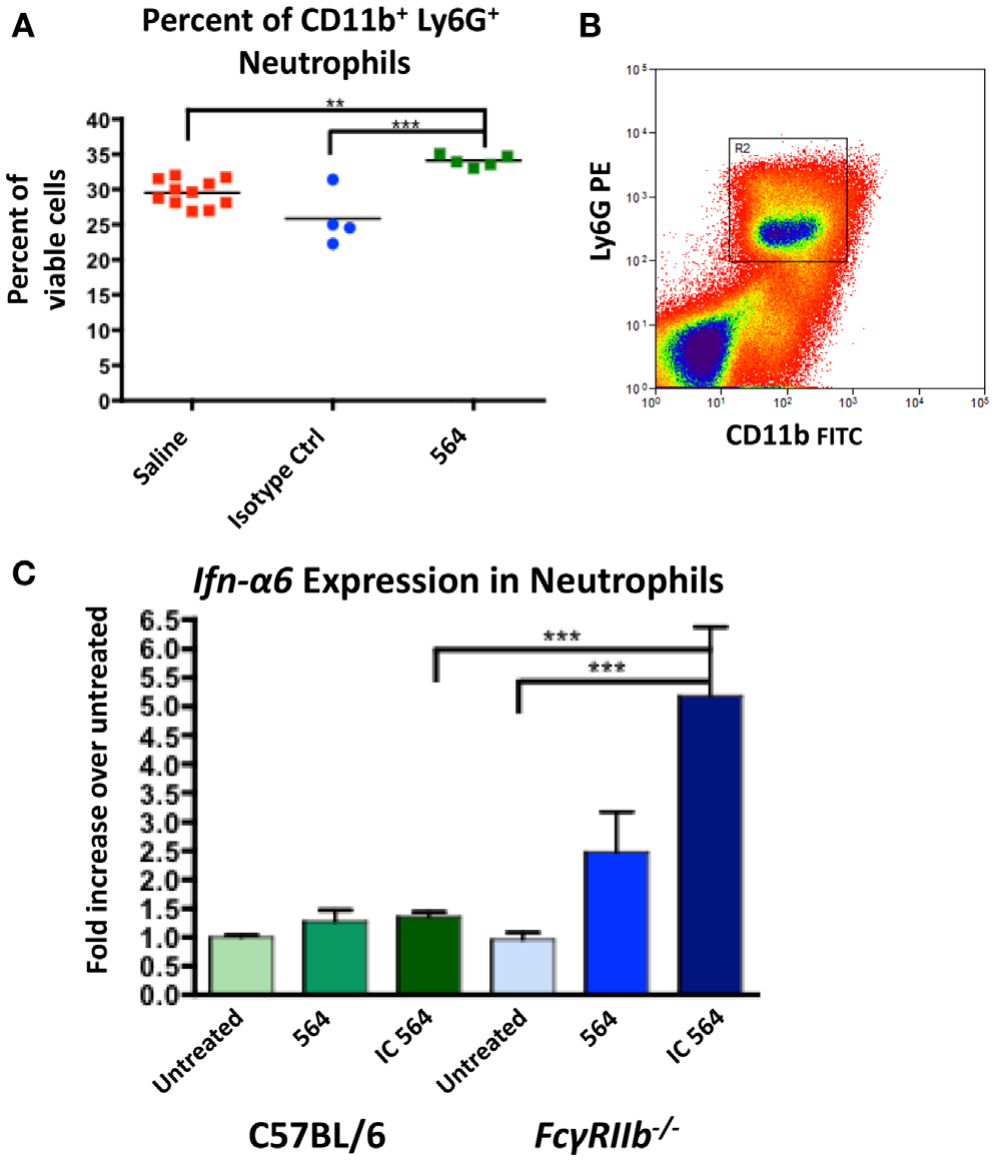
**564 IgG2b IC activates neutrophils to express *Ifn-*α*6***. **(A)** C57BL/6 mice were given intraperitoneal injections with 40 μg of 564 antibody, an isotype control of unknown specificity or a comparable volume of saline control. Two weeks later, the number of neutrophils in the bone marrow was determined by flow cytometry. Shown is the percent of neutrophils in each mouse. The average is shown as a horizontal line. **(B)** Neutrophils were purified from whole bone marrow cell suspensions from the indicated mice by cell sorting (CD11b^+^ Ly6G^+^). Shown is a representative plot of the gate used to purify neutrophils. **(C)** Purified neutrophils were cultured for 4 h in the presence of 0.01 μg/mL purified IgG2b 564 antibody or 564 IC made by incubating purified IgG2b 564 antibody with bone marrow-derived RNA. RNA was extracted from the cell culture, converted to cDNA, and *Ifn-*α*6* expression was measured by RT-qPCR. Expression was normalized to β*-actin* and compared to untreated samples from each genotype. Shown is the average ± SEM fold-increase in *Ifn-*α*6* expression in three mice per group tested in independent experiments, each done in triplicate.

564Igi mice were shown to have significantly more monocytes and neutrophils compared to C57BL/6 controls (30). To determine if the 564 antibody plays a role in the increase in granulocytes, we injected normal C57BL/6 mice with the 564 antibody and then whole bone marrow cell suspensions were analyzed for the presence of neutrophils 2 weeks later. The 564 antibody induced a significant increase in the percent of neutrophils in the bone marrow compared to a control saline injection and an isotype control (Figure 2A).

Both neutrophils and monocytes in 564Igi mice make significant contributions to IFN-I production (30). IFN-I signaling causes an upregulation of FcγRIV in 564Igi neutrophils, the activating receptor for IgG2a and IgG2b IC (30). The ratio of FcγRIV to FcγRIIb, the inhibitory receptor, sets the activation threshold for the response to IC (32). To test the neutrophil response to 564 IC, purified neutrophils from C57BL/6 mice (Figure 2B) were cultured *in vitro* in the presence of purified 564 antibody or 564 IC and *Ifn-*α*6* expression was measured by RT-qPCR. Previous studies have used *Ifn-*α*6* to study the role of endosomal TLRs in IFN-I production (15, 33). The 564 IC slightly increased *Ifn-*α*6* expression compared to untreated cells in C57BL/6 mice (Figure 2C, green), showing that the 564 antibodies in 564Igi mice may activate neutrophils. Furthermore, neutrophils from *Fc*γ*RIIb^−/−^* mice, which lack the inhibitory Fcγ receptor for IgG2b IC, show a significant increase in *Ifn-*α*6* expression (Figure 2C, blue), similar to the increased *Ifn-*α*6* expression in neutrophils that have upregulated FcγRIV (30). This suggests that altering the ratios of Fcγ receptors can change the sensitivity of neutrophils to IC. 564 IC did not have any effect on the expression of *Tlr7* and *Tlr8* in either C57BL/6 or *Fc*γ*RIIb^−/−^* neutrophils (data not shown). Purified 564 antibody also induced moderate *Ifn-*α*6* expression in *Fc*γ*RIIb^−/−^* neutrophils, likely due to the formation of IC *in vivo* after injection and the hyper-responsiveness of neutrophils from *Fc*γ*RIIb^−/−^* mice.

### TLR8 agonist induces *Ifn-α6* expression in neutrophils

**Figure 3:**
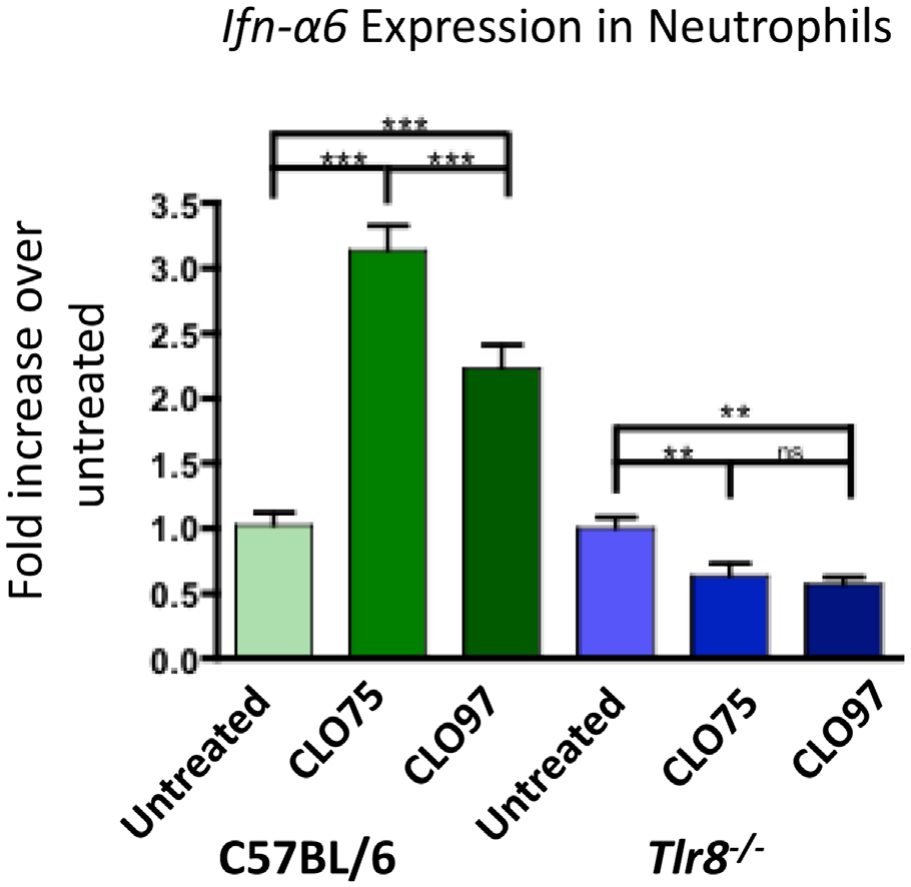
**TLR8 agonist induces increased *Ifn-*α*6* expression in neutrophils**. Neutrophils were purified from whole bone marrow cell suspensions from the indicated mice by cell sorting as in Figure 2B. Neutrophils were cultured for 16 h in the presence of the TLR8 agonist CLO75 (1 mg/mL) or the TLR7 agonist CLO97 (1 mg/mL). RNA was extracted from the cell culture, converted to cDNA, and *Ifn-*α*6* transcript level was measured by RT-qPCR. Expression was normalized to β*-actin* and compared to untreated samples from each genotype. Shown is the average ± SEM fold-increase in *Ifn-*α*6* expression in two mice per group tested in independent experiments, each done in triplicate.

To examine the role of *Tlr8* expression in IFNα production in neutrophils, we cultured purified neutrophils from C57BL/6 mice in the presence of the TLR8 agonist CLO75 or the TLR7 agonist CLO97 and measured *Ifn-*α*6* expression. Neutrophils from C57BL/6 mice treated with both CLO75 and CLO97 had increased *Ifn-*α*6* expression. However, CLO75 increased *Ifn-*α*6* significantly more than CLO97 (Figure 3, green). Neutrophils from *Tlr8*-deficient mice responded poorly to both CLO75 and CLO97 (Figure 3, blue). *Tlr7* expression is significantly decreased in *Tlr8*-deficient neutrophils (15), which may explain the poor response of *Tlr8*-deficient cells to a TLR7 agonist. Still, these data show that activation of TLR8 induces *Ifn-*α*6* expression in neutrophils.

### Bone marrow-derived macrophages from female mice over-express *Tlr8*, possibly through inefficient X-inactivation

**Figure 4:**
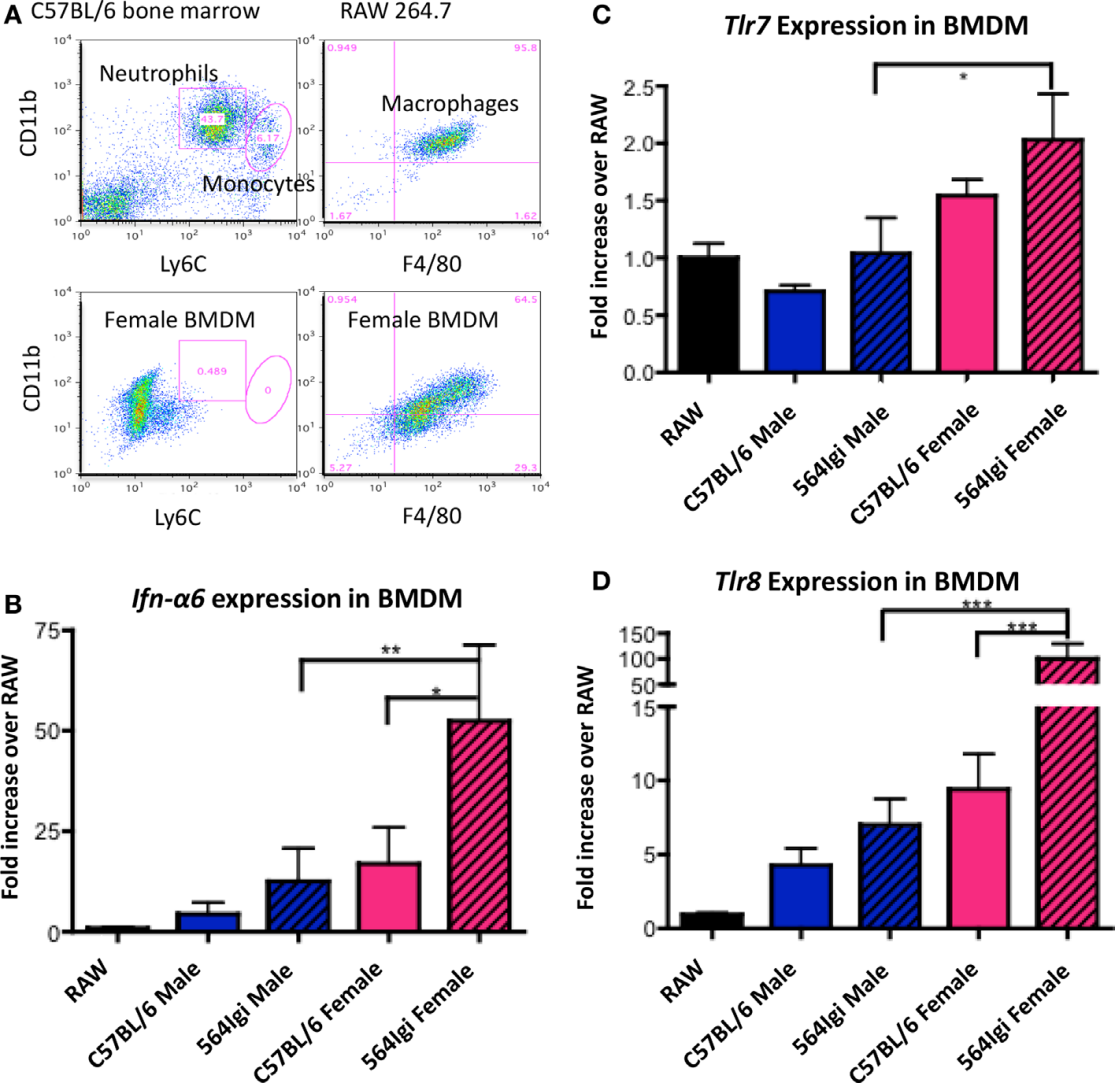
**BMDM from 564Igi females have increased transcript levels of *Ifn-*α*6*, *Tlr7*, and *Tlr8* compared to BMDM from male 564Igi mice**. **(A)** Whole bone marrow cell suspensions from female and male mice were grown in cell culture media to select for the growth of macrophages (20% FCS, 25% L-292 cell culture supernatant, 10 U/mL penicillin/streptomycin, RPMI). Shown is a representative plot showing the percentage of neutrophils, monocytes, and macrophages after culturing compared to whole bone marrow and RAW 264.7 macrophage cell line. **(B–D)** After 7 days in culture, RNA was isolated from the cells, converted to cDNA, and **(B)** *Ifn-*α*6*, **(C)** *Tlr7*, and **(D)** *Tlr8* expression was measured by RT-qPCR. Expression was normalized to β*-actin* and compared to *Ifn-*α*6, Tlr7*, and *Tlr8* expression in the RAW 264.7 macrophage cell line. Shown is the average ± SEM fold-increase in expression two mice per group, tested in two independent experiments, each done in triplicate.

As described above, 564Igi females have increased IgG autoantibodies compared to males (Figures 1A,B). Autoantibody production in 564Igi mice is mediated by TLR7 and TLR8 (15, 20). Therefore, the increase in pathogenic IgG autoantibodies in 564Igi females may be the result of differential expression of X-linked *Tlr7* and/or *Tlr8*. To examine the expression of *Tlr7* and *Tlr8*, we used RT-qPCR and RNA FISH. Neutrophils were not used in these experiments because neutrophils did not survive the culturing process necessary for RNA-FISH. However, we have previously shown that neutrophils from 564Igi mice over-express *Tlr7* and *Tlr8* compared to C57BL/6 mice at both the mRNA and protein level (15). Here, we use BMDM from male and female C57BL/6 and 564Igi mice because monocytes and macrophages were also shown to produce IFN-I in 564Igi mice (30). BMDM were cultured from whole bone marrow cell suspensions and analyzed by flow cytometry for phenotypic similarity to the RAW264.7 mouse macrophage cell line (Figure 4A). RAW264.7 was subsequently used for normalization in qRT-PCR analyses. BMDM from both C57BL/6 and 564Igi male and female mice express *Ifn-*α*6* (Figure 4B), similar to the primary neutrophils and monocytes/macrophages found in 564Igi mice (30). However, BMDM from female 564Igi mice express significantly more *Ifn-*α*6* than BMDM from male 564Igi mice and C57BL/6 female mice, suggesting that BMDM may significantly contribute to IFN-I production *in vivo*. The phenotypic similarities between BMDM and primary neutrophils/macrophages from 564Igi mice support our use of these cells in subsequent experiments. BMDM from female 564Igi mice also over-express *Tlr7* and *Tlr8* compared to male 564Igi-derived and C57BL/6-derived BMDM (Figures 4C,D). These results are similar to what has been published for primary neutrophils from 564Igi mice, which over-express *Tlr7* and *Tlr8* compared to neutrophils from C57BL/6 mice by both qRT-PCR and Western blot (15). Furthermore, the fold-increase in *Tlr8* expression in female BMDM is several orders of magnitude higher than the fold-increase in *Tlr7* expression. Although both *Tlr7* and *Tlr8* are located on the X chromosome, it appears that *Tlr8* may be more significantly dysregulated than *Tlr7* and may play a larger role in facilitating SLE pathogenesis in females.

**Figure 5:**
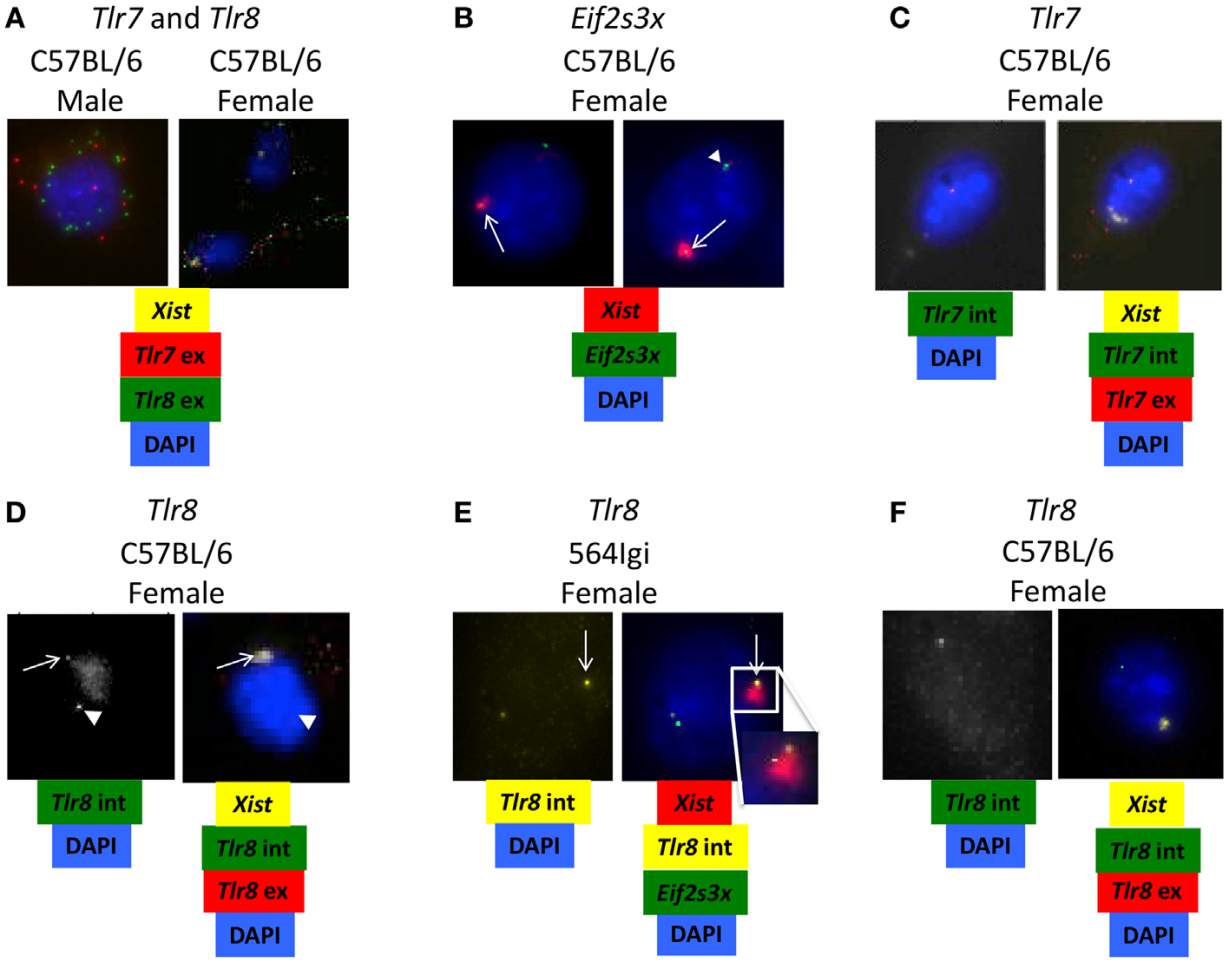
***Tlr8* RNA co-localizes with *Xist* RNA**. **(A–F)** BMDM from C57BL/6 and 564Igi mice were stained for the presence of *Xist* and **(A)** *Tlr7* and *Tlr8* exonic mRNA, **(B)** *Eif2s3x* intronic mRNA, **(C)** *Tlr7* intronic and exonic mRNA or **(D–F)** *Tlr8* intronic and exonic mRNA using fluorescent probes. **(A)** An example of increased *Tlr8* mRNA compared to *Tlr7* mRNA in female BMDM. **(B)** Two examples of escaped X-inactivation of *Eif2s3x*. **(C)** An example of normal X-inactivation of *Tlr7*. **(D)** An example of potential escaped X-inactivation of *Tlr8* in C57BL/6 female BMDM. **(E)** An example of potential escaped X-inactivation of *Tlr8* in 564Igi female BMDM. 1/800 cells (~0.1%) show this staining pattern. The insert shows the *Xist*-cloud with the *Tlr8* and weak *Eif2s3x* signals. **(F)** An example of normal X-inactivation of *Tlr8* in C57BL/6 female BMDM. The probes used in each set of images are shown underneath. Intronic (int) probes detect nuclear pre-mRNA before splicing. Exonic (ex) probes also detect cytoplasmic mature mRNA after splicing. White arrowheads show target RNA expression from active X chromosome. White arrows show target RNA expression from inactivated X chromosome.

It has been shown that 564Igi females with two normal copies of *Tlr8*, one on each X chromosome, express *Tlr8* approximately twofold higher than 564Igi females carrying only one copy of *Tlr8* (15). These data led to the hypothesis that inefficient X-inactivation may be responsible for the significant increase in *Tlr8* expression in female BMDM. To examine this possibility, we used RNA-FISH to detect the presence of *Tlr7* and *Tlr8* exonic mature mRNA in BMDM from C57BL/6 males and females. In female BMDM, there appears to be more *Tlr8* transcript than in male BMDM (Figure 5A), consistent with the RT-qPCR data (Figures 4C,D).

To determine if the increase in *Tlr8* expression is due to escaped X-inactivation, we used RNA-FISH to detect *Tlr7* and *Tlr8* immature intronic mRNA transcripts and *Xist* RNA, the long non-coding RNA required for X-inactivation (34) in C57BL/6 and 564Igi female BMDM. Co-localization of target intronic RNA and *Xist* RNA suggests that the gene is being expressed from the inactive X chromosome. *Eif2s3x*, a known essential gene with a high X-inactivation escape frequency (35), was used as a positive control (Figure 5B, top and bottom). We did not detect any cells with *Tlr7* intronic RNA and *Xist* RNA co-localized, suggesting that *Tlr7* is appropriately silenced on the inactivated X chromosome (Figure 5C), consistent with previous reports (36). However, there were some cells that showed *Tlr8* intronic RNA at two locations in the nuclei in both C57BL/6 and 564Igi BMDM: isolated from *Xist* RNA and co-localized with *Xist* RNA (Figures 5D,E), suggesting that *Tlr8* is being expressed from both the active and inactive X chromosomes in some cells. The transcription burst events for *Eif2s3x* and *Tlr8* seem uncoupled as we note expression from either or both alleles for both genes in some cells. In other cells, however, we capture the events for both alleles for both genes, with the burst from the inactive X chromosome located at the fringe of the *Xist*-cloud. Cells that showed co-localization of *Tlr8* and *Xist*, and hence *Tlr8* expression from both X chromosomes, also had more mature, exonic *Tlr8* mRNA transcript compared to cells that expressed a single copy of *Tlr8* (Figures 5D–F). Our data therefore are consistent with that *Tlr8* escapes X-inactivation in some macrophages, contributing to its higher expression in female-derived cells.

Our data suggest a model of SLE pathogenesis specific to females where *Tlr8* escapes X-inactivation, and, as a result, is over-expressed. Activation of TLR8 leads to *Ifn-a6* production in neutrophils. IFN-I can activate developing B cells (30) to promote the production of autoantibodies. The increased autoantibodies in circulation in female mice can then form IC that activate neutrophils and propagate the production of IFN-I and SLE pathogenesis.

## Discussion

Systemic lupus erythematosus has a strong gender bias with a 9:1 ratio of female:male patients. The mechanisms responsible for the female preference are unclear and may involve a combination of genetic and environmental factors. Here, we show that *Tlr8* escape of X-inactivation may be a driving factor for the increased incidence of SLE among female mice.

In addition to the pathogenic effects autoantibodies are known to exert in SLE patients, previous studies have shown that maternal autoantibodies are able to mediate developmental defects in offspring born to female SLE patients (3). Here, we show that female 564Igi mice have an increase in serum anti-RNA IgG2a and IgG2b antibodies (Figure 1A). Pregnant 564Igi female mice also yield litters with few females (Figure 1B), which seems to be a direct effect of circulating 564 antibodies (Figure 1C). IgG antibodies are able to cross the placenta during pregnancy and surpass maternal circulation levels in the newborn (37). In particular, neurological defects are common in offspring born to SLE mothers (6–11, 38). Although the blood–brain barrier prevents antibody access to the adult brain, the immature blood–brain barrier in developing embryos allows antibodies to enter the developing brain (37). Studies have confirmed that some classes of anti-DNA antibodies are able to react with fetal neuronal receptors and cause neuronal death via excitotoxicity and female fetal death (7, 12–14). The receptor is expressed at different times during development in male and female embryos, which may explain why the defect is largely seen in only female offspring (14). The 564 antibody also reacts with a consensus peptide sequence of the neuronal receptor (data not shown). Therefore, circulating 564 antibodies in 564Igi female mice may mediate female fetal death by a similar mechanism. Furthermore, these data show that anti-RNA antibodies, not only anti-DNA antibodies, are pathogenic to developing offspring.

We have previously shown that *Ifn-*α*6* expression in neutrophils of 564Igi mice is dependent on TLR8 *in vivo* (15). Those results are confirmed here in C57BL/6-derived cells *in vitro* showing that cells treated with a TLR8 agonist induce *Ifn-*α*6* expression (Figure 3). In addition, IFN-I signaling in 564Igi mice upregulates FcγRIV expression in neutrophils (30), which may lower the activation threshold by increasing the FcγRIV:FcγRIIb ratio. Here, we directly show that 564 antibodies cause an expansion of neutrophil populations (Figure 2A) and induce neutrophils to express *Ifn-*α*6* (Figure 2C, green). Furthermore, the absence of FcγRIIb dramatically increases *Ifn-*α*6* expression, presumably by infinitely increasing the FcγRIV:FcγRIIb ratio (Figure 2C, blue). IFN-I has been shown to activate immature B cells in the bone marrow of 564Igi mice (30). Additionally, IgG autoantibodies in 564Igi mice are known to be the result of CSR during early B cell development in the bone marrow (39). Thus, the self-reactive B cells in the bone marrow of 564Igi females may be stimulated with more IFN-I and produce more anti-RNA IgG2a and IgG2b antibodies. These antibodies may then feedback as IC to activate more neutrophils, whose activation threshold may have been lowered due to the increased FcγRIV:FcγRIIb ratio, further promoting IFN-I production, and ultimately disease pathogenesis.

Previous studies using the 564Igi mouse model of SLE on a *Tlr7/9^−/−^* background show that gene dosage of X-linked *Tlr8* plays a significant role in disease pathogenesis. Female 564Igi *Tlr7/9^−/−^* mice with two normal X chromosomes, and hence two functional copies of *Tlr8*, produce autoantibodies, express *Ifn-I* in neutrophils, have expanded populations of granulocytes, and ultimately develop glomerulonephritis (15). Male 564Igi *Tlr7/9^−/−^* mice, on the other hand, only have one X chromosome and, therefore, only one copy of *Tlr8*. Male mice do not produce autoantibodies, have low expression of *Ifn-I* in neutrophils and fewer granulocytes than female mice (15). To distinguish between hormonal differences and gene dosage effects as potential causes for the differences between male and female phenotypes, female 564Igi *Tlr7/9^−/−^* mice were generated with one X chromosome lacking *Tlr8*. These female mice were genetically similar to their male counterparts in that they expressed only one copy of *Tlr8* while retaining normal female hormone levels. Autoantibody production, *Ifn-I* expression and granulopoiesis were all diminished in 564Igi *Tlr7/9^−/−^* females with only one copy of *Tlr8* (15). These results suggest that differences in gene dosage of *Tlr8*, not hormonal differences, are responsible for the SLE-like phenotype in 564Igi *Tlr7/9^−/−^* females.

To more closely examine the mechanisms of *Tlr8* expression in female mice, we analyzed the level of X-inactivation of both *Tlr7* and *Tlr8* in C57BL/6 and 564Igi female BMDM. *Tlr8* intronic mRNA can be found in two locations in the nuclei of some BMDM: isolated from *Xist* RNA and co-localized with *Xist* RNA (Figures 5D,E), suggesting that *Tlr8* is expressed from both the active and the inactivated X chromosomes. At least 15% of human genes consistently escape X-inactivation, with an additional 10% of genes escaping X-inactivation in a tissue- or cell-specific manner (40). These genes largely cluster to the distal portion of the X chromosome short arm (Xp) (40). *Tlr7* and *Tlr8* are also located at the distal end of Xp. One model of X-inactivation spreading proposes that long interspersed repeat elements (L1) serve as *cis*-acting elements to promote inactivation (41). L1 concentrations are inversely correlated with the proportion of genes that escape inactivation along the chromosome; the most distal portion of Xp has the most genes that escape activation and the lowest concentration of L1 (40, 42). This provides a mechanism by which *Tlr8* may inappropriately escape X-inactivation: although both *Tlr7* and *Tlr8* are located at the distal end of Xp, *Tlr7* may be in close proximity to an L1 element, ensuring its appropriate inactivation. *Tlr8*, on the other hand, may be further from an L1 element, allowing its escape from X-inactivation in some cells.

Toll-like receptor 8, like TLR7, is an intracellular sensor of single-stranded RNA. Under normal circumstances, TLRs do not recognize self nucleic acids. However, it has been shown that self-RNA or -DNA can trigger cell activation when transported to TLR-containing endosomes (43–45). Endogenous retroviral nucleic acid products have also been implicated in SLE by activation of TLRs. High concentrations of retroviral envelope glycoprotein gp70 have been found in the sera of (NZB x NZW)F1, MRL, and BXSB lupus-prone mouse models upon immune activation (46). However, there have been few studies analyzing the expression of endogenous retroviral products between males and females in SLE. Anti-gp70 immune responses depend on TLR7, suggesting that the recognition of retroviral RNA initiates the response (24, 47). Unlike TLR7, little is known about murine TLR8 because, until recently, it was believed to be non-functional. Therefore, TLR8 may also play a role in the induction of immune responses to retroviral RNA. The upregulation of *Tlr8* in BMDM from female 564Igi mice (Figure 4D) may make cells more sensitive to endogenous RNA products and induce cells to express *Ifn-I*. An alternate explanation is that female mice express more retroviral RNA, stimulating a more robust TLR8 response due to its higher expression.

There is a long history of endosomal TLR involvement in autoimmune disease. Studies of TLR7, 8, and 9 generally used universal knockout models, so the specific roles of these TLRs in B cells and myeloid cells remain unclear. Recent studies have shown that the protective and pathogenic effects of TLR9 and TLR7, respectively, on systemic inflammation are due to B cell-intrinsic effects (48, 49). One model used in those studies resulted in 20% of myeloid cells also being deficient for TLR7 or TLR9 (48). Whether the small population of TLR-deficient myeloid cells contributed to the phenotype of the mice remains unknown. However, it seems clear that spontaneous germinal center formation requires B cell-intrinsic *Tlr7* expression and is negatively regulated by B cell-intrinsic *Tlr9* expression (49). Here, we show that TLR8, which has not been examined in a B cell-specific manner, may escape X-inactivate in myeloid cells and contribute to the elevated IFN-I production and systemic inflammation seen in the 564Igi mouse model of SLE. The precise roles of endosomal TLRs in autoimmune disease are still being investigated and, dissecting the differences between lymphoid- and myeloid-intrinsic factors will be critical to understanding these roles.

Collectively, our data show that inefficient X-inactivation of *Tlr8* in female mice may contribute to the significant gender bias of SLE. The resulting increase in *Tlr8* expression then facilitates downstream pathologies, including IFN-I production by neutrophils and autoantibody production, ultimately creating a positive feedback loop to propagate IFN-I production and disease pathogenesis.

## Conflict of Interest Statement

The authors declare that the research was conducted in the absence of any commercial or financial relationships that could be construed as a potential conflict of interest.

## Funding

This work was supported by the National Institutes of Health grants R01AI45104 and R01AI076409A. We are also grateful for generous support from the Esche Fund and the Keck Foundation.
